# From video summarization to real time video summarization in smart cities and beyond: A survey

**DOI:** 10.3389/fdata.2022.1106776

**Published:** 2023-01-09

**Authors:** Prashant Giridhar Shambharkar, Ruchi Goel

**Affiliations:** Department of Computer Science and Engineering, Delhi Technological University, New Delhi, India

**Keywords:** computer vision, video summarization, real time video summarization (RVS), keyframes, summary

## Abstract

With the massive expansion of videos on the internet, searching through millions of them has become quite challenging. Smartphones, recording devices, and file sharing are all examples of ways to capture massive amounts of real time video. In smart cities, there are many surveillance cameras, which has created a massive volume of video data whose indexing, retrieval, and administration is a difficult problem. Exploring such results takes time and degrades the user experience. In this case, video summarization is extremely useful. Video summarization allows for the efficient storing, retrieval, and browsing of huge amounts of information from video without sacrificing key features. This article presents a classification and analysis of video summarization approaches, with a focus on real-time video summarization (RVS) domain techniques that can be used to summarize videos. The current study will be useful in integrating essential research findings and data for quick reference, laying the preliminaries, and investigating prospective research directions. A variety of practical uses, including aberrant detection in a video surveillance system, have made successful use of video summarization in smart cities.

## 1. Introduction

Analyzing video content to extract valuable or intriguing information is difficult and time-consuming. Many videos are uploaded to YouTube, IMDB, tourism sites, Flickr, and other video-sharing sites every minute. Every minute, 300 h of video are posted to the YouTube channel and about one billion hours of video are watched every day (You Tube Stats, n.d). Millennial video cameras are installed in smart cities including public spaces, public transportation, banks, airfields, and other locations, resulting in a tremendous amount of data that is difficult to analyze in real time. There will be hundreds of suggestions for each search topic; navigating through these lengthy videos to find the essential video takes time, and also challenging to efficiently obtain this much data in a short amount of time.

Secondly, due to the abundance of videos, users must rely on metadata such as title, image, description, and remark to locate the video they want to see. This metadata, too, isn't a trustworthy predictor of the accompanying video's semantic content, leaving consumers with no choice but to skim it to get a general idea of what's going on. To address these concerns, work is underway to construct a video summary that summarizes the entire video in a short period. A video comprises a series of video frames. If a video summary has a high recall, precision, and low redundancy rate, it is deemed to be good (Tiwari and Bhatnagar, 2021). When a video is played, each frame is presented in a specific order and at a specific frame rate. According to the Cisco Visual Networking Index published in March 2020, video and other applications are still in high demand in smart cities, but future application requirements will result in large bandwidth demands, even beyond the prediction period of 2023. Video summarization is a technique for quickly exploring large collections of videos, as well as indexing and accessing content more efficiently. The generated video summaries may vary depending on the application, and the same video may have multiple summaries depending on the user's or application domain's requirements (Senthil Murugan et al., 2018). The concept of video summarization is to make exploring a huge collection of video data faster and more efficient, as well as to achieve efficient access and representation of video content (Li et al., 2003).

Depending on how video data is accessed, processing can be done in either the compressed or uncompressed domain. Some of the characteristics given by conventional video encoders are included in compressed domain approaches. An uncompressed summary, on the other hand, makes use of all of the information in the frames (Iparraguirre and Delrieux, 2014).

### 1.1. Need for summarization

Smart cities face various complicated challenges from managing transportation networks to securing people to enhancing emergency response times. Smart camera video data provides a rich, time-based record of urban surroundings, but its sheer volume and complexity make it challenging to analyze and use. It is necessary to provide smart cities with fast and accurate information to increase efficiency and quality of life. The volume of digital video data has expanded dramatically in recent years due to the growing use of multimedia applications in domains such as education, entertainment, commerce, and medicine (Ajmal et al., 2012). Smart video can collect rich data in almost real-time, these datasets can also be very large, expensive to transmit and store, and labor- and time-intensive to analyze. Secondly, This task is far more complex than analyzing text documents because of the video's multimodal character, which sends a wide range of semantics in various formats, including sound, music, static images, moving images, and text (Dimitrova, 2004). The enormous video data must be managed correctly and efficiently to maximize the usability of these huge recordings. As a result, video summarization is an important and rapidly expanding study field. Users may manage and explore large videos more effectively and efficiently with the help of a video summary (Yasmin et al., 2021). This research aims to identify and establish the video summarizing approaches that have been discovered in the literature, with a particular emphasis on real-time video summarization. The phrase “real time” refers to the amount of elapsed time to summarize a video that is smaller than the original video's duration. RVS aids in the indexing and retrieval of videos from a library. It also aids the consumer in deciding whether or not to view the complete video (Bhaumik et al., 2017).

### 1.2. Challenges

Due to the computational complexity, creating a good video summary in real time while retaining subjective quality is difficult. Overall, video summarization poses several difficulties. Some of them are as follows:

1) One of the most challenging parts of video summary is subjectivity; because various annotators may have different perspectives, different people may select different important shots for the same video (Otani et al., 2017). It is difficult for them to agree on what is relevant and what is not.
2) Jitter effects caused by the camera wearer's movement, are more difficult to summarize. Use cases for smart cities include sophisticated traffic monitoring, legal parking management, speed detection. Additional problems with lifelogging video summaries include accurate feature tracking, uniform sampling, and large data streams with very narrow limits (Mahesh Kini and Pai, 2019).
3) Collecting summarization labels takes time, and a little dataset will not be sufficient. Because the available datasets only contain videos of specific sorts, the model performs poorly on videos from other categories. To deal with this, we can use unsupervised, semi-supervised, or multi-task learning. Computed hardware and development complexity are well-known issues (Del Molino et al., 2017).
4) The Internet of Things is rapidly evolving, and it is quickly displacing traditional sensing in existing systems. Vision sensors in IoT have lately been popular due to their widespread use in smart cities and industries for a variety of applications, including security. Various mechanisms can be offered for analyzing observed personnel in the industry for aberrant activity and generating alerts (Hussain et al., 2020).
5) Deep neural networks, which require a significant amount of labeled data to train, are used in modern video summarizing approaches. Existing datasets for video summarization, on the other hand, are small-scale, which makes deep models readily overfit.

The rest of the paper follows the structure as; Section 2 gives an introduction to the video summarization and its types. The scope and approach of RVS are described in Section 3. Section 4 covers the systematic review of real time video summarization. Section 5 is the prominent study for RVS. The next sections include a conclusion and references.

## 2. Video summarization

Video data is today's most generated data. A group of representative video frames (e.g., video key-frames) or video fragments (e.g., video key-fragments) sewed in progressive sequence to generate a shorter video is commonly used to create the produced summary. Video summarization aims to provide an overview of the content that highlights the video's most instructive and relevant elements.

### 2.1. Video summarization steps

**Figure 1:**
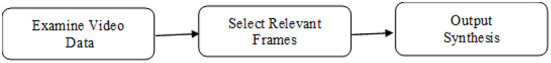
Steps to video summarization.

To identify which parts of videos are to be removed, video summarization algorithms must rely on video content. There are three main steps to video summarization (Mahesh Kini and Pai, 2019) as indicated in Figure 1. The first step is to examine video data to determine the most important features, structures, or processes within the components visual, audio, and textual (audio and textual component if exists). The second step is to select relevant frames that represent the video's content, and the third step is to output synthesis, which involves assembling the frames/shots into the original video.

### 2.2. Video summarization techniques

The criteria for relevant frame selection (score prediction and keyframe selection) may differ for different users and application domains, a general framework for a video summarizing will not work for everyone. There are many approaches to video summarization as indicated in Table 1.

**Table 1:** Approaches of video summarization.

**Approach type**	**SubTypes**	
Summary based	1. Static	
	2. Dynamic	
	3. Hierarchical	
	4. Multi-view	
	5. Image	
	6. Text	
Preference based	1. Domain-specific	
	2. Query based	2.1 Generic
		2.2 Query Focused
	3. Semantic based	
	4. Event based	
	5. Feature based	
Domain based	1. Pixel	
	2. Compressed	
Information source based	1. Internal	
	2. External	
	3. Hybrid	
Time based	1. Real time	
	2. Static	
Training strategy based	1. Supervised	
	2. Unsupervised	
	3. Semisupervised	

### 2.2.1. Summary based

Summary based video summarization can be further classified into static, dynamic, hierarchical, multi-view, image, and text summaries (De Avila et al., 2011).

*2.2.1.1. Static summary* is also known as keyframing or storyboard presentation. It's a montage of keyframes taken from the authentic video. A static summary is more suitable used for indexing, browsing, and retrieval (Cahuina and Chavez, 2013). To assess video static summaries De Avila et al. (2011) used two-stream deep learning architecture that combined the k-means clustering algorithm with color information collected from video frames.

*2.2.1.2. Dynamic summary* is also known as Video Skimming. Short dynamic sections (video skims) or audio and the most relevant video shots are chosen. The goal of video skimming techniques is to choose pictures or scenes from the full video and compile them into a relevant summary. Zhong et al. (2019) offer a novel dynamic video summarization approach.

In static summaries, the motion component is lost. However, the technology makes video storage and retrieval easier, particularly in large video repositories. Storyboard layouts lack audio cues and may lack continuity, yet they are efficient in terms of calculation time and memory.

*2.2.1.3. Hierarchical summary* represents a scalable and multi-level summary. It has several levels of abstraction, with the highest level having the fewest keyframes and the lowest level having the most. V-unit (Ren and Jiang, 2009) for shot detection is used to structure videos following the hierarchical model to remove junk frames.

*2.2.1.4. MVS (MultiView Summary)* considers multiple points of view at the same time and creates a representative summary of all of them. Smart IIoT-based architecture with an embedded vision for detecting incredulous articles, and exchanging traffic volume statistics are proposed by the author (Hussain et al., 2021).

*2.2.1.5. Image summary* A single image or a collection of images is typically used for this type of summary. Images serve as synopsis rather than frames or shot. Authors (Trieu et al., 2020) presented a paradigm that extends the single-image image captioning transformer-based architecture to multi-image captioning.

*2.2.1.6. Text summary* These are summaries that consist solely of a paragraph-length textual summary of a video sequence. It is created utilizing Natural Language Processing (NLP) techniques and does not include any audio or visual descriptions. Text summaries are cost-effective in terms of storage and calculation, but they are unable to communicate all of the information since they lack the audio and visual components of the video sequence.

#### 2.2.2. Preference based

It is broadly divided into 5 categories listed Video summarization is domain-specific, query-based, semantically based, event-based, and feature-based.

*2.2.2.1. Domain-specific* Kaushal et al. (2019) provide a summary based on what is relevant to that domain, as well as other desirable domain features like representativeness, coverage, and diversity, for a given video of that domain.

*2.2.2.2. Query-focused* aims to create a diversified selection of video frames or segments that are both connected to the query and contain the original video data. While customizing video summarizers appears to be a promising direction (Sharghi et al., 2017). It can be divided into two categories (Xiao et al., 2020): (1) Generic and (2) Query-focused.

In the case of Generic video summarization, when a substantial scene transition occurs in a video, a broad summary is constructed by choosing keyframes. The keyframes are extracted when the cluster number for a frame changes. The visual components of the video are extracted using a pre-trained Convolutional Neural Network (CNN), which is then clustered using k-means, and a sequential keyframe-generating procedure follows.

*2.2.2.3. Semantic-based* These are summaries that are generated based on the video's content and are mostly based on objects, actions, activities, and events with a high level of interpretation based on domain expertise (Phadikar et al., 2018; Jiang et al., 2019).

*2.2.2.4. Event-based* the objective is to develop and maintain succinct and coherent summaries that describe the current state of an event. Event-based video summarization is preferred over key frame-based summarization for surveillance video summarization. Many different applications use intelligent video surveillance systems to track events and conduct activity analysis (Chauhan and Vegad, 2022).

*2.2.2.5. Feature-based*: Motion, color, dynamic contents, gesture, audio-visual, voice transcripts, objects, and other factors are used to classify feature-based video summarization techniques. Apostolidis et al. (2021) used relevant literature on deep-learning-based video summarization and covered protocol aspects for summary evaluation.

#### 2.2.3. Domain based

It can be divided into pixels and compressed.

*2.2.3.1. Pixel domain* video summarization works by collecting information from the pixels of the frames in a video to summarize it. In most applications, a video is compressed, and decoding the video to summarize it takes a lot of time and space.

*2.2.3.2. Compressed domain* video summarization includes extracting features from compressed video by partially decoding it, which solves this problem. Fei et al. (2018) devised a method for compressing a shot's most significant activities into a single keyframe in a compressed video stream in the Compressed Domain. It can provide a brief and colorful summary of video information. The original footage is represented by many keyframes created from one rich keyframe from each shot. Phadikar et al. (2018) proposed a DCT (Discrete Cosine Transform) compressed domain image retrieval scheme. A feature set was created using edge histograms, color histograms, and moments. The best feature vector is then constructed using GA.

#### 2.2.4. Information source based

It is further classified as internal, external, or hybrid. At various phases of the video lifecycle, a video summarizing algorithm evaluates a range of information sources to abstract the semantics associated with a video stream's content and then extract the various audio-visual cues. Based on the information sources they examine, the various methodologies reported in the literature can be divided into three groups (Money and Agius, 2008):

*2.2.4.1 Internal*: Examine internal data extracted from the video stream generated during the video lifecycle's production step. These methods extract semiotics from a video stream's image, audio, and text at low-level data for use in a video summary.

*2.2.4.2 External*: To look at data that isn't generated right away from the video stream, external summarization approaches are used. External information can be divided into two types: Contextual (not directly from a user's point of view) and User-based information (derived from a user's direct input). For Contextual Hussein et al. (2019) presented a video graph that is used to simulate the long-term temporal structure of human activities. The semantic gap that internal summary approaches confront can be solved using external summarization techniques.

*2.2.4.3 Hybrid*: During any point of the video lifecycle, hybrid summarization algorithms examine both the movie's internal and external data. Hybrid summarization algorithms can leverage the semantics of the text to a greater extent, resulting in higher levels of semantic abstraction. This method is very well suited to summarizing domain-specific data. Kanehira et al. (2018) devised a broad video summarizing approach that seeks to estimate the underlying perspective by taking video-level similarity into account, which is supposed to be obtained from the related viewpoint.

#### 2.2.5. Time based

Depending on whether or not it is done on a live video, summarization can be classified as real-time or static as discussed in section 3, or on a video recording, respectively.

*2.2.5.1. Real-time* In these circumstances, selecting crucial frames while the video is being captured depending on the context of the video will be quite valuable. It's challenging to sum up a video in real time because the output needs to be supplied quickly. In real-time systems, a late output is a bad output.

*2.2.5.2. Static based* A-frame from the unified collection of frames collected from the source video is used to show the input video in a static summary (Nair and Mohan, 2021).

The most crucial elements of the original video are included in keyframes, which are a subset of frames.

#### 2.2.6. Training strategy based

Due to insufficient feature extraction and model selection, machine learning-based approaches can occasionally result in poor video summary quality. For example, a model with too few features may be inaccurate, whereas a model with too many features may be overfitted (Gygli et al., 2014). The following are some broad categories for a deep-learning-based video summarizing algorithms: Supervised approaches, Unsupervised approaches, and Semi-supervised approaches (Apostolidis et al., 2021). The summary should keep keyframes from the original video. The same frames may be important for some at the same time and uninteresting for another viewer thus, making a video summary a highly subjective word (Gorokhovatskyi et al., 2020).

*2.2.6.1. Supervised* Theses approaches are used to train a model using labeled data before generating video summaries. Deep neural networks have recently been used in video summarization. The temporal information is extracted using recurrent neural networks (Zhao and Xing, 2014). For each movie, these supervised approaches necessitate a huge number of frame or shot-level labels. As a result, gathering many annotated films is expensive.

*2.2.6.2. Unsupervised* There are no labeled data samples available in an unsupervised approach, so the frames are classified into several categories based on content similarity. Fajtl et al. (2019) propose a new soft attention-based simple network for sequence-to-sequence transformation, which is more efficient and less difficult than the current Bi-LSTM-based encoder-decoder network with soft attention. In an unsupervised manner, a deep summarizer network is used to reduce the distance between training films and the distribution of their summarizations. A summarizer like this can then be used to estimate the best synopsis for a new video (Cooharojananone et al., 2010).

*2.2.6.3. Semi-supervised approach* This contains both labeled and unlabeled data. This mixture will often have a small bit of labeled data and a significant amount of unlabeled data.

## 3. Real time video summarization

Whether a live stream on a personal blog or a security camera in a manufacturing facility, video data is a common asset used daily. Real-time image and video processing involve producing output while simultaneously processing input. The typical frame rate is connected to real-time image and video processing.

The current capturing standard is typically 30 frames per second. To process all the frames in real time, they would have to be processed as soon as they were captured.

So, if the capture rate is 30 frames per second, 30 frames must be processed in 1 s.

Existing methods for a video summarizing generally take either an offline (Gygli et al. 2014) or an online (Zhao and Xing, 2014) approach. To generate a summary, offline techniques require knowledge of and access to the complete video stream. Such solutions, on the other hand, necessitate storing the entire video at the same time, which is resource costly and/or unfeasible (for example, for unboundedly long video streams).

Alternatives to the aforementioned include online or streaming video summarizing tools. An online summarization method takes a video stream and generates a summary on the go and at any time as the data stream elements come, without relying on any future data. Because they simply maintain a small piece of the previous video stream (or information related to it) in memory, such approaches can be made to use less memory. This situation is particularly interesting because online methods are computationally less expensive than their batch counterparts when batch processing a video is too resource-intensive on a device, when an application needs access to the historical summary, or for unboundedly long video streams.

Unlike offline options, generating a video summary online comes with several challenges and is significantly more difficult due to insufficient video information. The video summary's quality may be harmed by the short delay, progressive generation, and absence of the complete video sequence information (e.g., content and length) (Almeida et al., 2013).

**Figure 2:**
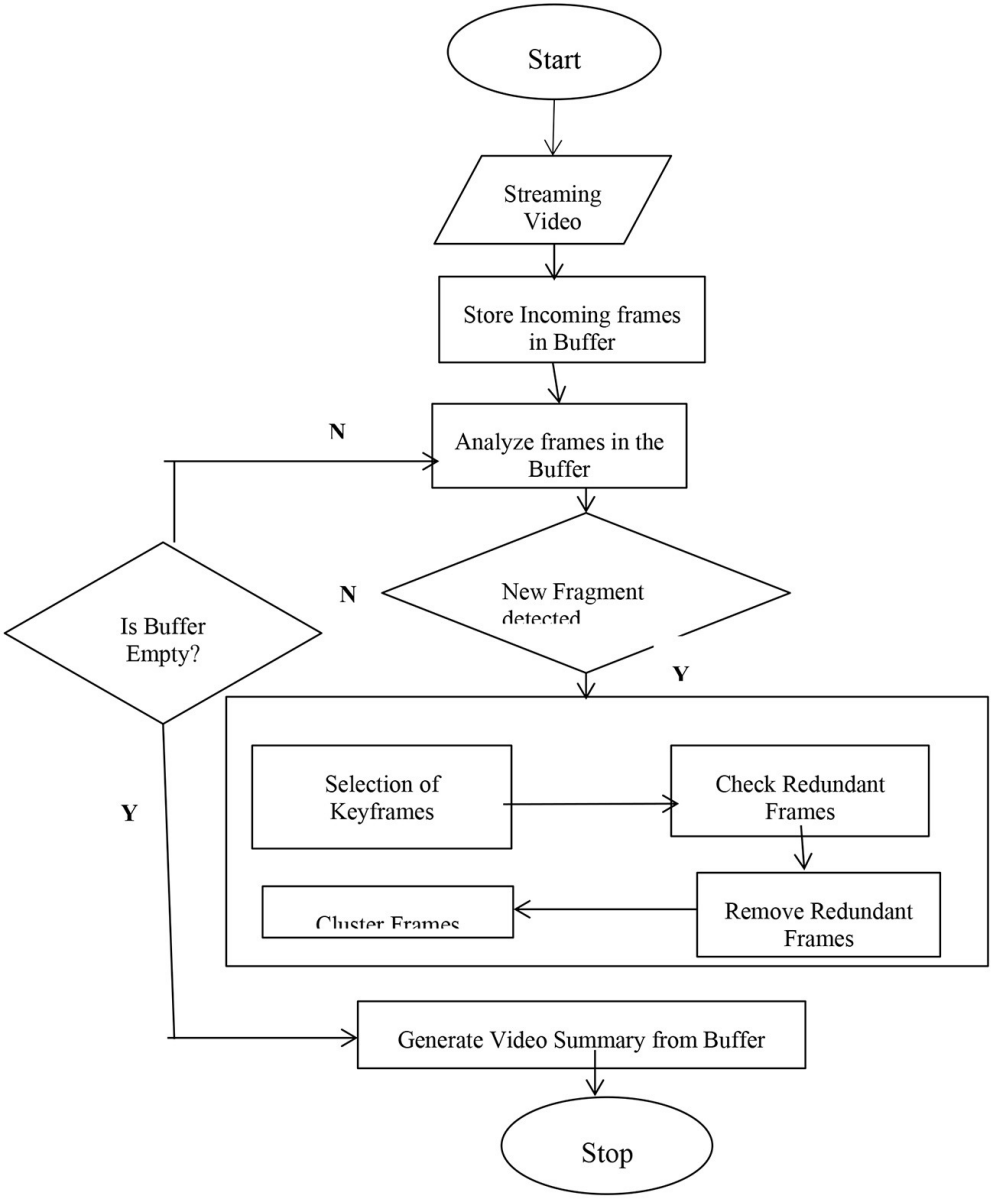
Flowchart of real time video summarization.

The flow chart of Real Time Video Summarization is shown in Figure 2. The video's incoming image frames are gathered in a buffer (BIF) for subsequent analysis. If the image frames arrive in non-sequential order, they must be sorted. This is done to keep the frames temporal relationship and make further processing easier. Before the feature extraction procedure, the incoming frames in the buffer are continually read. After that redundant frames are checked and if found those frames are removed. Clustering/scoring of important frames is done, and a final summary is generated.

### 3.1. Problem formulation of real-time video summarization

Let V be the number of frames of video in real time.


(1) $$\begin{array}{l} V=\left\{F_{1},F_{2},F_{3}-------------Fn\right\} \end{array}$$

Then summarized video in real time RVs is a collection of all relevant frames from Video V.


(2) $$\begin{array}{l} RV_{\text{S}}=\{R_{\text{i}1},R_{\text{i}2},R_{\text{i}3}-------------R_{\text{tx}}|\\ i=1tot,x<<n\} \end{array}$$

For calculating the keyframes, the start and end frame numbers are recorded. It is easy to see that in a time-sequenced group of image frames, the best representative frame will be chosen that most accurately convey the core of the sequence.

## 4. Systematic review

**Figure 3:**
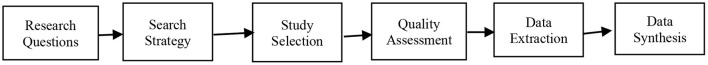
Review process.

A systematic review is a study of a specific issue that employs systematic and repeatable techniques for categorizing, critically evaluating, and selecting all relevant research, as well as gathering and analyzing data from the studies included in the analysis. It was adopted for performing the study of Ketchenham and Charters' systematic literature review format (Kitchenham et al., 2009). The analysis process flow diagram is given in Figure 3. The review process was broken down into six stages viz. “research questions formulation, search strategy, study selection, quality assessment, data extraction, and data synthesis.” The first phase's goal was to formulate the research questions (RQs) related to video summarization. The second stage strategy for a comprehensive search was established and a good number of appropriate papers for detailed analysis were listed. Then the scope of this research is limited to include applicable studies in this area using Inclusion Exclusion Criteria. Ensured that the research included is consistent and similar and that the study's restrictions are well defined. The worthiness of the selected studies is evaluated in the quality assessment phase. The purpose was to determine the boundaries of review and ensure quality. Then, based on the Literature Survey, data is extracted to answer the research questions.

### 4.1. Research questions

From the study of the literature review following RQs were identified:

- RQ1: What methodology does the real time video summarization method employ?
- RQ2: What applications require real-time performance, and which do not?
- RQ3: To identify whether the video summarizing should be done in real-time or off-line.
- RQ4: On which datasets real time video summarizations performed?

### 4.2. Search strategy

This phase is intended to classify the appropriate collection of research papers for analysis. It involves an effective study strategy published in the last 15 years, i.e., from April 2006-to-date. The search terms for this SLR were real time video summarization and online video summarization. Popular digital libraries (publishers) research papers include Springer, ACM, IEEE, Science Direct, Wiley, Taylor, and Francis. The only title was considered in the title. Consequently, the main aim of this phase was to define and collect all the relevant research papers needed to perform a review.

### 4.3. Study selection

This method isolates the outdated, redundant, and unsuitable studies based on the 'Exclusion-Inclusion' selection criterion. It performs the filtration process by either selecting or rejecting studies that directly within the specified problem domain promote or address at least one research question (RQ). The following conditions for Inclusion-Exclusion are followed.

**Inclusion criteria:**

- The research is focused on the field of real-time video summarization.
- The research gives a well-described summary method or real time video summarization.
- Studies carried out over the last 15 years i.e. from April 2006-till date.

**Exclusion criteria:**

- Studies that lack adequate empirical or comparative analyses.
- Studies including video summarization detection on languages other than English (Languages like Bengali, Chinese, and Spanish, for example, are not covered).
- Books and works of gray literature.
- A research study that is duplicated.
- The study's entire text is unavailable.

### 4.4. Quality assessment

The selected studies were analyzed during this process to determine the importance and intensity of the studies chosen. A quality review has already been ensured because only high-quality, high-impact journals from reputable digital libraries were evaluated.

### 4.5. Data extraction

This step summarized and extracted information from the selected study based on mapping to one or more RQs. Information such as authors, year of publication, datasets used, methods, strengths, and weaknesses were the details from the extracted research studies. All of this information was then placed in a table that was used to synthesize data.

### 4.6. Data synthesis

The purpose of this step is to summarize and interpret the information obtained. In this review, the extracted data is summarized in tabular form and presented using different visual methods like graphs, charts, etc. The search terms found were input as a search query and applied to the selected digital libraries resulting in 15 articles.

## 5. Literature survey

In this section, a brief idea about all the selected studies has been given in reverse chronological order in the form of a table. The state-of-the-art is presented in Table 2 where all primary studies are succinctly reviewed according to publication year, author, the dataset used, method, strength, and weakness of the suggested technique of the literature (SLR) used during this process to examine the extensive research found in the description of opinions and their fields of application. Table 2 lists the preliminary research from the literature reviews.

**Table 2:** Prominent studies on real time or online summarization.

**S.No**	**References**	**Dataset**	**Method**	**Strength**	**Weakness**
1.	Ren and Jiang (2009)	TRECVID'08	Activity-level summarization, hierarchical modeling, and adaptive clustering	Secure, successful, and quick processing of compressed domain	Junk frames can be removed
2.	Cooharojananone et al. (2010)	TRECVID (London Gatwick surveillance video)	DSCD (Direct Shift Collision Detection)	Removing the non-essential information from the pool of summary data	Compression ratio is raised at such intervals, the summarized video is unable to compress any further.
3.	Almeida et al. (2012)	Open Video and youtube video	feature extraction; content selection; and noise filtering	Proposed strategy for allowing users to customize their own experience	User feedback can be used to refine summary
4.	Almeida et al. (2013)	TRECVID 2007	feature extraction; content selection; and noise filtering	A speedy and high-quality summary is generated	Similarity Metrics can be used to reduce a video summary
5.	Zhao and Xing (2014)	YouTube and real-world surveillance videos	YouTube and real-world surveillance videos	Group sparse coding to Create a dictionary from a video	Upgraded the viewer to understand the summarized video without the use/requirement of LiveLight
6.	Wang et al. (2014)	Columbia Consumer Video's UGVs	Columbia Consumer Video's UGVs	Subjective evaluation	The semantics of the video have been identified
7.	Ou et al. (2015)	26 videos from own surveillance system	26 videos from own surveillance system	Gaussian mixture model	Wireless video sensors are proposed as part of a system to assist save energy.
8.	Marvaniya et al. (2016)	SumMe	SumMe	Dictionary learning	The dictionary update efficiency has been achieved.
9.	Choudhary et al. (2017)	SumMe and SumLive	Shot detection and scoring are combined	Shot detection and scoring are combined	Analysis of footage captured by a live camera and real time summary is generated
10.	Yousefi et al. (2018)	ADL	Semantic information	Online control charts applied for identifying	Memory issue
11.	Taylor and Qureshi (2018)	SumMe	LSTM method	The approach could be suitable for mobile deployment	Feature computation for each segment is limited
12	Lal et al. (2019)	Youtube and TVSum/SumMe datasets	Convolutional LSTM	Provide a concise and diverse summary	Next frame loss higher
13	Ghani et al. (2019)	Open- video.org's public video dataset	Clustering	Keeping redundancy frames to a bare minimum in terms of storage space	Can be applied to HD videos
14	Zhang M. et al. (2020)	MIT-Adobe 5K	CNN	Computational cost saved	Unable to remove pixel-level noise
15	Jain et al. (2021)	COCO	Mask R-CNN	Ability to effectively describe videos in real time	Long videos are not summarized effectively

**Figure 4:**
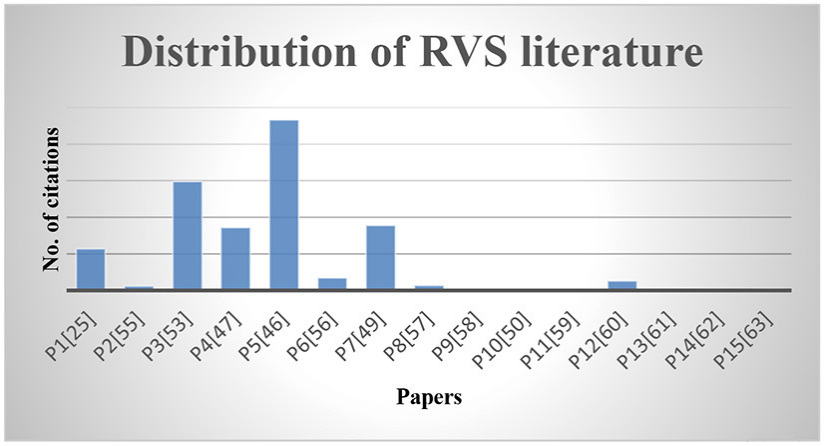
Distribution of RVS literature.

The distribution of RVS literature along with the citation of each paper is shown in Figure 4.

### 5.1. RQ1: What methodology does the real time video summarization method employ?

The rapid rise of video data and lack of time has necessitated the deployment of effective and advanced video summarizing (Vasudevan and Sellappa Gounder, 2021). Most deep-learning-based video summarization algorithms gather visual information from video frames using deep feature vectors obtained using pre-trained neural networks. After training, the Deep Summarizer Network's output can be a static video storyboard made up of selected video frames (key-frames), or brief video skims made up of video-chosen fragments (key fragments).

Graph-based clustering approaches were used to construct a discontinuous contour evolution and keyframe extraction-based method for summarizing movies in real time (Calic et al., 2008). The information from the complete video should be used to provide a better synopsis. Only earlier video frames can be accessible in the online video summary (Ou et al., 2015).

Two factors must be considered when selecting a descriptor for an online application: high representation ability and cheap computing cost (Yousefi et al., 2018).

An existing video summarizing approach typically reduces the input video in one of three ways: Within frames choose keyframes, key sub-shots, and key objects.

For time-sensitive applications like sports, filtering live content in real-time is extremely useful. The system recognizes and filters critical occurrences in live broadcast video programs before adjusting the rate of incoming streams based on the material's importance. The value of a feature can be determined by the structure of the software and the preferences of the users (Zhong et al., 2001; Yadav et al., 2020) suggested a deep neural network for generating natural language descriptions and abstractive text summaries of a live video series input. The text summary is generated by picking keyframes from videos and using those keyframes for picture captioning.

The keyframes, according to Zhang Y. et al. (2020) must capture the motions of essential objects. Online auto-encoding, which can discover spare and salient object motion clips, was used to recover, and analyze the trajectories of moving object instances.

Almeida et al. (2012) offer VISON, a new compressed-domain video summarizing approach. It summarizes the video content by combining visual cues from the video stream with a simple, fast algorithm. Users can adjust the quality of the video summary based on how eagerly people anticipate VISON.

RT-Venet was offered by Zhang M. et al. (2020) as a way to improve high-resolution videos in real-time. Although generative CNNs with the fundamental encoder-decoder structure have shown good image-to-image translation results, they are not appropriate for real-time enhancing tasks.

RCEA (Zhang T. et al., 2020) is proposed to help annotate emotions when watching mobile videos without adding to the user's mental load. For automatic real-time video summarization, researchers have traditionally used unsupervised approaches. This can be done by looking at the entire video or noticing the slight differences between adjacent frames.

For video summarization (Yadav et al., 2020), employs LSTM, a supervised approach based on RNN. In the summarizing challenge, the context of a video captioning mode is employed to build a more semantically oriented video representation.

The author (Jain et al., 2021) proposed a technique for effectively summarizing videos in real time. To identify common objects, the Mask R-CNN model trained on the COCO dataset is employed. The objects seen in the video frame have been precisely marked. Lastly, a video summary is created by combining all of the annotations.

### 5.2. RQ2: What applications require real-time performance, and which do not?

Various image and video processing applications require real-time (online), while others require offline processing. Because the source frame rate and resolution determine the processing time for each frame, real-time algorithms cannot be overly complicated.

New hardware solutions now enable quicker processing speeds. However, depending on the application, there are still certain limits. Real-time feedback and processed images from sensors are required for a variety of real-time applications, including traffic monitoring, military target tracking, observation, and monitoring, real-time video games, and other programs. Real-time video enhancement, which aims to improve the visual quality of live videos, can be used in video communication, augmented reality, and robotics applications. A real-time video summarization system can process videos online, eliminating the possibility of backlogs.

Offline processing allows for more complicated and computationally intensive algorithms, yielding better results than real-time processing. Processing an already recorded video sequence or image offline is called post-processing (Online and Offline Processing, n.d.).

Real-time processing, on the other hand, is required in some applications. For example, programs that require real-time feedback and processed images from sensors include traffic monitoring, target tracking in military applications, surveillance and monitoring, and real-time video games.

**Figure 5:**
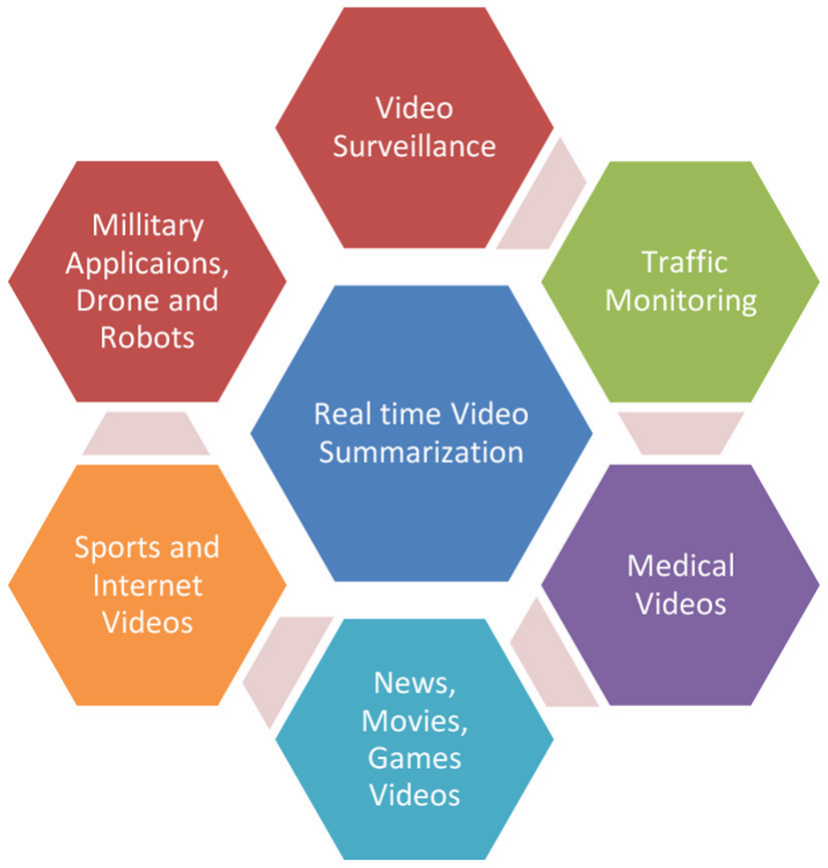
Applications of real time video summarization.

Since the processing time for each frame is defined by the source frame rate and resolution, real-time algorithms do not have the luxury of great complexity. New hardware solutions are now available that provide faster processing speeds, but there are still constraints that apply depending on the application. Figure 5 shows the application of real time video summarization in different fields.

### 5.3. RQ3: To identify whether the video summarizing should be done in real-time or offline

Video is a reliable source of information, and video usage has exploded in recent years, both online and offline. Unfortunately, most known video summarizing algorithms work offline, which implies that the summation process can only begin once the complete video has been captured. Additionally, the computational complexity and memory resource requirements are often high, making them unsuitable for many applications, which is why online summarization is used. A solution that performs both efficiently and gradually is required for the online production of a video summary.

**Figure 6:**
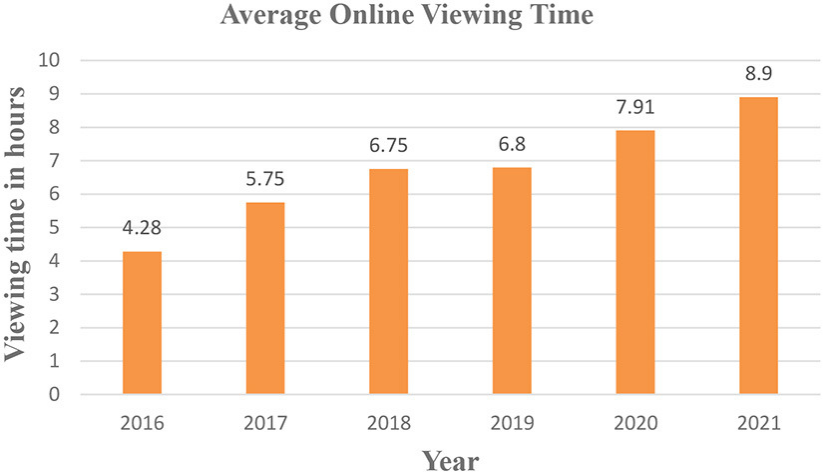
Average online viewing time.

The popularity of online video continues to rise at the expense of traditional broadcast viewing, as shown in Figure 6. Viewers spend approximately 8 h (7 h and 55 min) each week on average, consuming a variety of entertainment. The viewing duration increased significantly in 2021, with an average of 8.9 h, 7.91 in 2020, up 16 percent from 2019, 6.8 h, and 85 percent from 2016', with 4.28 average online video hours.

### 5.4. RQ4: On which datasets are real-time video summarizations performed?

Even though video summarizing approaches have been intensively investigated, no standard protocols or heuristic ideas for assessing their efficacy exist.

**Figure 7:**
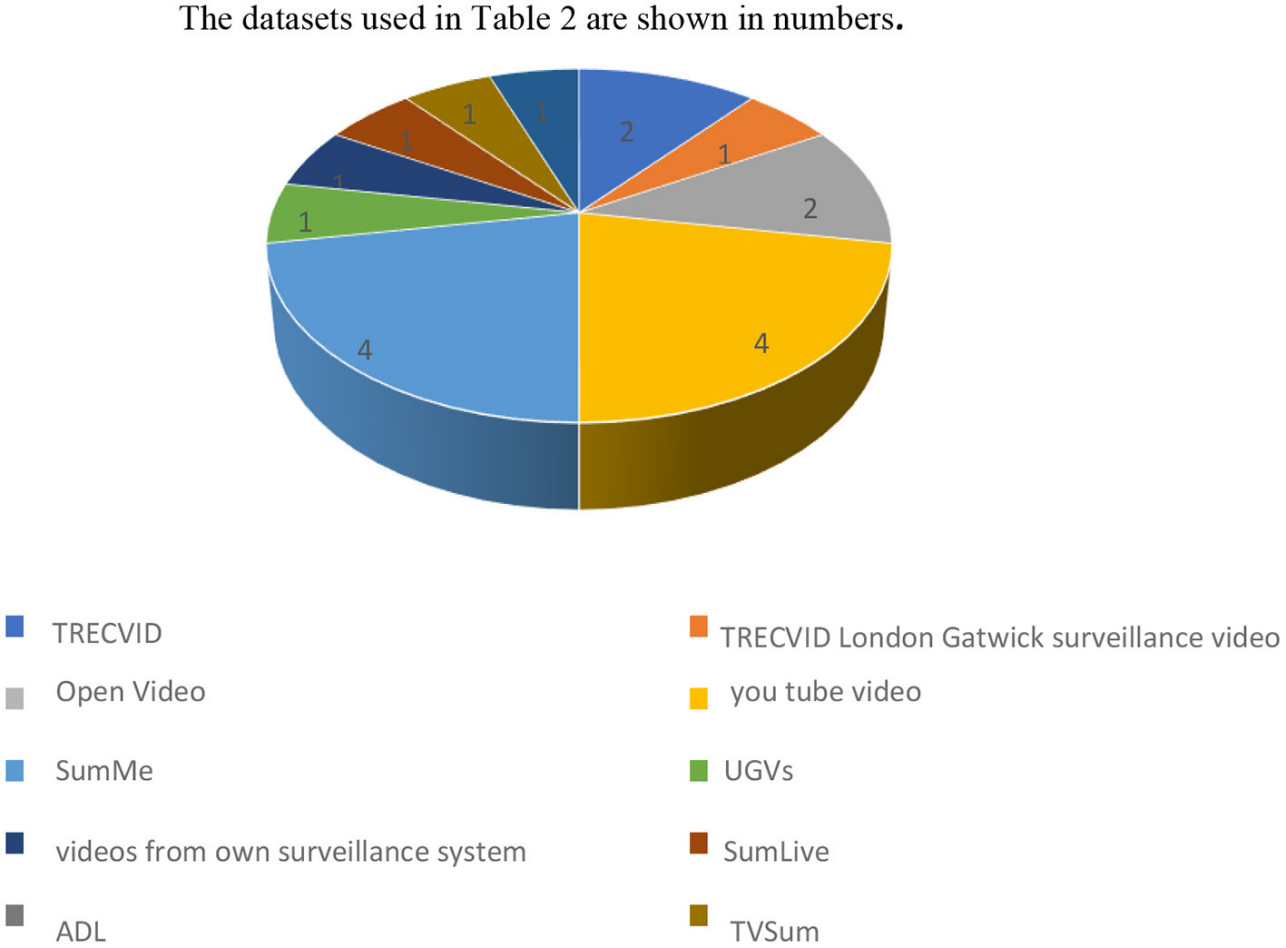
Dataset used in real time summarization.

Because summary evaluation is a subjective process, manual comparisons are difficult to achieve reliable results. In the video summarizing bibliography, many datasets stand out: SumMe (Choudhary et al., 2017; Taylor and Qureshi, 2018), TVSum, ADL (Yousefi et al., 2018), TRECKVID'08 (Ren and Jiang, 2009), and COCO (Jain et al., 2021) rushes require a thorough video summary. The dataset used in Table 2 is shown in Figure 7. It has been studied how many researchers have worked on which dataset. The number of studies that have used that specific dataset is depicted in a graph plotted based on the analysis. SumMe is a collection of 25 short videos that range from 1 to 6 min and cover various topics such as vacations, festivals, and sports. SumLive is made up of 15 films ranging from 2 to 6 min. The f- measure was used to evaluate the algorithm (Gygli et al., 2014). Many researchers have used a benchmark database for video retrieval, summarization, and indexing, such as TREC Video Retrieval Evaluation (TRECVID) (Naphade and Smith, 2004).

Trec Video Dataset is a dataset that reports rush videos and helps in video summarization. In TRECKVID 08 (Ren and Jiang, 2009) rushes necessitate a thorough video description, the clustering of retakes of the same scene, and the removal of junk footage.

Zhang M. et al. (2020) use the MIT-Adobe 5K dataset, which consists of 500 source photos. Five different exporters used are classified as (A/B….).

### 5.5. Challenges and future directions for research in real time video summarization

Real Time-based video summarizing approaches are helpful in practical applications such as security video summarization, sports video highlights, military applications, aerospace, medical, computer, financial, and many more. The viewpoint and perspective of summarization are frequently application dependent. Semantic understanding and representation are the most critical difficulties for incorporating diversity in video and human perception. A single failure can lead to a catastrophic system failure in fault-tolerant computing. Another area within networking where real time is relevant is quality of service (QoS) because bad QoS can lead to customer defection, among other reasons. This article has covered real time video summarization. Compared to different summarization approaches, real time video summarization is a less explored issue. Users often want tailored video summaries that reflect their specific video interests in less time and space. Many computer vision problems have been solved using deep learning approaches. Video summarization systems will have to progress in the future to generate user-specific compressed video summaries with excellent efficiency in less time. GPU computation has grown in prominence due to its ability to solve various computer vision issues. Because of the inherent parallel processing and the large video dataset that must be dealt with, real time video summarization can be expedited by utilizing GPUs.

## 6. Conclusion

Due to its value and importance in many domains, video summarization research is developing rapidly. This paper thoroughly analyzes current real-time video summarization (RVS) methodologies and discovers less research in the RVS. For training their systems, most approaches employed datasets from OVP, YouTube, and SumMe/TVSum. RVS is in demand today and is used in many applications. Working in real-time and preventing problems before they develop benefits the bottom line by lowering risk and improving accuracy. A real-time video summarizing system might process videos in real time, effectively removing the backlog risk. New algorithms for more accurate keyframe extraction, better trash frame elimination, and more intriguing content inclusion could be implemented to improve the situation even further. Smartness is utilizing data and technology to improve decisions and quality of life. Despite advancements in Internet technologies, dynamic real-time data distribution to responders continues to be a concern. Even if the devices collect data in real time from various sources, the device is only helpful if the processed data in the form of a video summary is precisely generated or covers all keyframes. Due to this, it will be challenging to handle emergencies and critical circumstances. Because of the widespread use of live video applications, real-time video improvement is in high demand in smart cities. Still, present techniques need to meet the stringent requirements for speed and reliability.

## Data availability statement

The original contributions presented in the study are included in the article/supplementary material, further inquiries can be directed to the corresponding author.

## Author contributions

RG: research work. PS: supervision. All authors contributed to the article and approved the submitted version.
